# Heparanase inhibition prevents glycocalyx damage and albuminuria in experimental minimal change disease

**DOI:** 10.1002/ctm2.70706

**Published:** 2026-05-31

**Authors:** Michael Crompton, Raina D. Ramnath, Kai Betteridge, Sara Desideri, Aldara Martin Alonso, Lulu Jiang, Monica Gamez, Holly Stowell‐Connolly, Viktoriia Vasylchenko, Karen L. Onions, Matthew J. Butler, Chris R. Neal, Andrew H. J. Salmon, Jeremy E. Turnbull, Hiroshi Kawachi, Olga V. Zubkova, Gavin I. Welsh, Simon C. Satchell, Rebecca R. Foster

**Affiliations:** ^1^ Bristol Renal Bristol Medical School University of Bristol Bristol UK; ^2^ Copenhagen Centre for Glycomics Institute of Cellular and Molecular Medicine University of Copenhagen Copenhagen Denmark; ^3^ Department of Cell Biology Institute of Nephrology Niigata University Graduate School of Medical and Dental Sciences Niigata Japan; ^4^ Ferrier Research Institute Victoria University of Wellington Wellington New Zealand

1

Dear Editor,

In this work, we show that targeting heparanase therapeutically in a model of minimal change disease (MCD) restores the vascular endothelial barrier and protects against albuminuria, independently of podocyte slit‐diaphragm damage.

MCD is a kidney disease defined by minimal glomerular structural changes by light microscopy, yet sudden onset nephrotic‐range albuminuria. Ninety percent of nephrotic syndrome is caused by MCD in children and up to 25% in adults.^1^ Up to 50% of patients with nephrotic range albuminuria who do not respond to treatment, progress to end stage renal disease within 8 year of diagnosis.^2^

MCD is associated with injury to the specialized epithelial cell of the glomerular filtration barrier (GFB), the podocyte, evidenced by podocyte foot process effacement, only measurable by electron microscopy.^3^ The podocyte governs filtration control through the specialized protein complex between foot processes; the slit diaphragm. Treatments that target glomerular structural changes are urgently needed in MCD.

The luminal side of the GFB is coated with an endothelial glycocalyx (eGlx), damage to which is associated with albuminuria. This glycoprotein, proteoglycan and glycolipid‐rich layer contains the glycosaminoglycan, heparan sulphate (HS). Glomerular heparanase has been shown to be upregulated in glomerular disease.^4^ Increased circulating heparanase activity and HS fragments have been demonstrated in MCD patients, indicating glomerular eGlx damage, associated with albuminuria.^5^ Since the eGlx is the first part of the barrier to albumin in the GFB, we hypothesized that eGlx is damaged in MCD as a consequence of slit diaphragm dysregulation, leading to upregulated heparanase and albuminuria.

We demonstrated this using a well‐established rat model of MCD and a novel class of water‐soluble heparanase inhibitor (HI), OVZ/HS‐1635^6^; a synthesized tetravalent HS glycomimetic, capped with sulphated maltose disaccharides, with 23 nM IC_50_ potency for heparanase inhibition. Female Lewis rats were tail‐vein injected with a single dose of 5 mg of mAb 5‐1‐6, which targets the extracellular domain of the rat homolog of nephrin (a critical part of the podocyte slit‐diaphragm),^7^ or vehicle (saline). Anti‐nephrin antibodies (AN‐Ab) are present in 61% of MCD patients with active disease, but are absent in the same patients when disease is inactive, suggesting they have a role in the pathogenesis of disease.^8^ Thus, our rat model of anti‐nephrin antibody (AN‐Ab) nephropathy mimics the human form of disease ensuring the relevance of this model. AN‐Ab nephropathy induced changes in podocin distribution, without any loss of podocytes as shown by immuno‐staining of the podocyte transcription factor, Wilms Tumour‐1 (WT‐1) (Figure S1A–Ciii). Nephrin mRNA expression was reduced, and severe albuminuria occurred by Day 3 (Figure S1D,E). Systolic blood pressure was unaffected by AN‐Ab at endpoint (sham: 114 ± 12, AN‐Ab: 110 ± 11 mmHg). Together, these data demonstrate that AN‐Ab nephropathy induces albuminuria via targeting nephrin in podocytes, in a blood pressure independent manner.

Ultrastructural podocyte changes were quantified by electron microscopy, showing significantly increased foot‐process width and reduced slit‐diaphragm width, suggestive of podocyte foot‐process effacement (Figure 1A–C), as anticipated. There was no change in podocyte glycocalyx depth, basement membrane thickness or glomerular endothelial fenestration number (Figure 1D–F). However, there was a significant reduction in glomerular eGlx (Figure 1G) and increased urinary heparanase (Figure 2Ai–iii), mirroring what was previously surmised in patients.^5^ Heparanase cleaves HS from its core protein and is processed from an inactive 65 kDa pro‐enzyme to an active 50 and 8 kDa heterodimer which is necessary for HS cleavage. Active urinary heparanase was only significantly increased at Day 3, but glomerular heparanase mRNA expression did not change at endpoint‐ day 7 (Figure 2Aiii,B), potentially having transiently increased and returned to baseline. It is also conceivable that urinary heparanase was derived from circulating haemopoietic or inflammatory cells.

**FIGURE 1:**
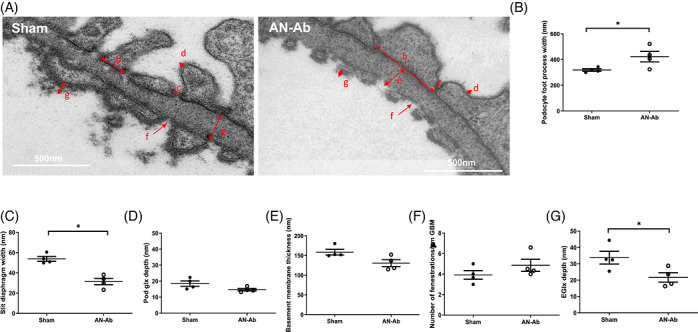
AN‐Ab induced ultrastructural changes to the glomerular filtration barrier. Rats given mAb‐5‐1‐6 or vehicle for 6 days were whole body perfusion fixed with glutaraldehyde in the presence of Alcian blue to image the endothelial glycocalyx (eGlx). Representative electron microscopy images are shown for sham and AN‐Ab nephropathy rats (A). Ultrastructural changes were measured as indicated on for; podocyte foot process width (b), summarized in B; slit diaphragm width (c), summarized in C; podocyte glycocalyx depth (d) summarized in (D); glomerular basement membrane (GBM) thickness (e), summarized in E; number of endothelial fenestrations per µM GBM (f), summarized in F: eGlx depth (g), summarized in (G). Mann Whitney test, one tailed, *n *= 4. * = *p* < 0.05.

**FIGURE 2:**
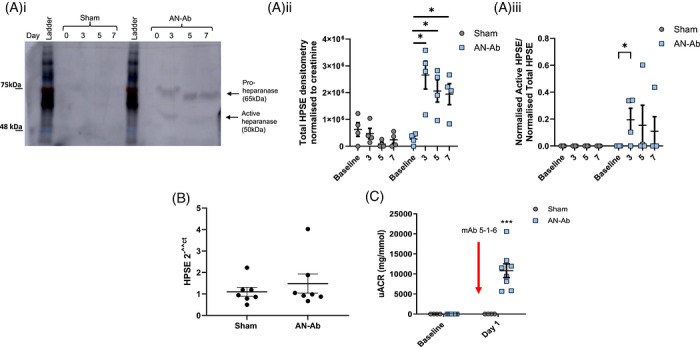
AN‐Ab increases availability of total and active heparanase. Glomeruli were sieved from rats given mAb 5‐1‐6, or left untreated, for 6 days and mRNA was extracted for QPCR. (Ai) Urine was western blotted and probed for heparanase which detects total (65 kDa) and active (50 kDa) bands. A representative blot for 0, 3, 5 and 7 days is shown for sham and AN‐Ab rats. (Aii) Densitometry is shown for total heparanase normalized to urinary creatinine (*n* = 4 two‐way ANOVA, * = *p* < 0.05), Bonferroni's multiple comparison test indicated) and for active heparanase nomalized to total heparanase (Aiii, *n* = 4 two‐way ANOVA, * = *p* < 0.05), Bonferroni's multiple comparison test indicated). (B) Glomerular heparanase (HPSE) mRNA was quantified in sham and AN‐Ab rats, normalized to house‐keeping gene and expressed relative to sham (2^−^^CT^). (C) uACR at day 1, before intervention, is shown with mAb 5‐1‐6 treatment indicated (a combination of both day 1 mAb 5‐1‐6 groups shown in (B), *n* = 4‐8, two‐way ANOVA, *p* < 0.001).

Of note, AN‐Ab causes severe albuminuria by Day 1 (Figure 2C), therefore intervention given after this point would be clinically relevant. In a second cohort, to confirm whether glomerular eGlx loss and albuminuria were dependent on heparanase, rats were injected with AN‐Ab (Day 0) and then treated daily, i.p. with HI (20 mg/kg, Day 1 as previously in pre‐clinical studies),^6^, ^9^ until Day 7 (Figure 3A). There was no significant effect on weight between the treatment groups (Figure 3B). Longitudinal data shows that urine‐albumin‐creatinine ratio (uACR) continued to increase significantly in untreated AN‐Ab, but not once HI treatment began (Figure 3 Ci). We suspect that the AN‐Ab insult was too severe for the rats to recover completely. Albuminuria was significantly reduced ∼60% by HI treatment at endpoint (uACR AN‐Ab: 17,483 ± 5269 vs. AN‐Ab + HI: 7250 ± 2176 mg/mmol, Figure 3Cii), glomerular eGlx depth was significantly restored (Figure 3Di–iii) demonstrated by glomerular capillary luminal lectin depth by immunofluorescence and confocal microscopy^10^ and there was a significant inverse correlation between the two (Figure 3E). Nephrin immunofluorescence across groups demonstrated significant loss of nephrin linearity in the rats with reduced albuminuria (Figure S2A,B), suggesting that improvement of albuminuria was independent of the original podocyte injury. This report is limited to using a single animal model. Future work could test effectiveness of HI in other models of MCD (e.g., puromycin aminonucleoside) to demonstrate common mechanism.

**FIGURE 3:**
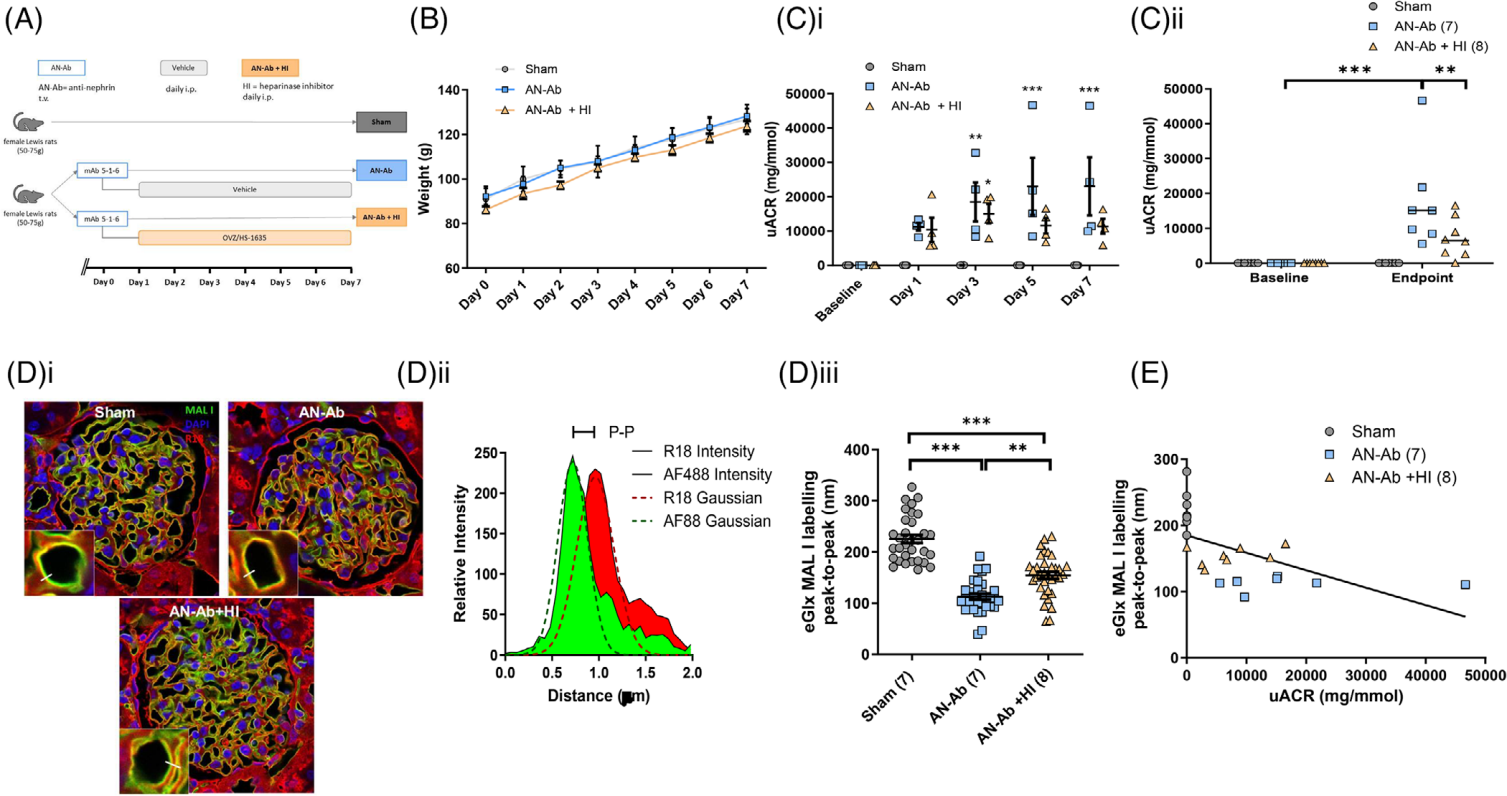
Targeting heparanase reduces proteinuria and preserves eGlx in AN‐Ab nephropathy. (A) Schematic to show experimental design of female Lewis rats with AN‐Ab induced or left untreated. Of those AN‐Ab rats, half were given heparanase inhibitor (HI) from Day 1 (daily) and half were given vehicle (sham). **(B)** End point uACR data is shown (*n* = 6–8, two‐way ANOVA, *p* < 0.001). (C) Rats were perfused with Ringer's solution. Kidneys were removed and paraffin embedded and stained with Alexa Fluor conjugated 488 MAL‐1 lectin to bind to glycosaminoglycans, octadecyl rhodamine B chloride (R18), to label the capillary wall and DAPI (nuclear counterstain). A linear region of interest (ROI) is drawn across the capillary lumen, such that all lectin staining inside the capillary lumen defines the eGlx (Ci). An example of the fluorophore profiles from the ROI are shown (green = lectin, red = R18) and the distance from one fluorophore peak to the other was quantified as a measure of eGlx depth (Cii). EGlx depth is shown, summarized between treatment groups (Friedman test (*p* < 0.0001) with Bonferroni's multiple comparison test). Statistics carried out on number of animals (shown in brackets). Mean glomerular values presented. (D) Endpoint uACR and eGlx depth from each animal were significantly correlated (linear regression, *r* ^2 ^= 0.32, *p* < 0.006).

We have previously shown that OVZ/16‐35 can rescue albuminuria in a model of type 2 diabetes, whilst protecting the glomerular eGlx,^9^ suggesting common mechanisms for albuminuria. Heparanase is an attractive drug target as it is essentially inactive in healthy conditions. Other HI closely mimic the properties of heparin, a natural HI. which can result in undesired effects such as anticoagulation. OVZ/16‐35 is a tetravalent dendrimer HS glycomimetic, which demonstrates no anticoagulant activity.^6^ Our work suggests this inhibitor has great potential to protect against eGlx damage and hence albuminuria in MCD. Ongoing studies are now focussed on pharmacokinetics, bioavailability and long‐term safety to support progression of OVZ/16‐35 towards clinical evaluation.

Together, these data challenge the dogma that MCD treatment is dependent upon podocyte health, thus increasing potential therapeutic targets and may lead to a change in management of these patients. To conclude, this work demonstrates that glomerular endothelial glycocalyx damage is a therapeutic target in patients with MCD, particularly those with nephrin autoantibodies, and it provides a novel class of heparanase inhibitor suitable for clinical use.

## AUTHOR CONTRIBUTIONS

M.C., R.D.R., K.B., S.D., A.M.A., L.J. and M.G.—all made substantial contributions to conceptual design, acquisition of data and interpretation of data. H.S‐C., V.V. and K.L.O.—made substantial contributions to acquisition and interpretation of data. M.J.B., C.N., A.S., J.E.T., H.K., O.V.Z., G.I.W., S.C.S. and R.R.F. made substantial contributions to conceptual design and interpretation of data. R.R.F. wrote the draft and is accountable for all aspects of work. All authors revised the manuscript and gave their final approval.

## CONFLICT OF INTEREST STATEMENT

Olga V. Zubkova and Jeremy E. Turnbull are named inventors on patents NZ 712476 and US11,186,603B2 (Heparan sulfate glycomimetic compounds and their pharmaceutical and cosmeceutical uses) which relate to the compound (OVZ/16‐35 or Tet‐29) described in this manuscript. The other authors declare no conflicts of interest.

## FUNDING INFORMATION

M.C., R.D.R., K.O., M.G.: British Heart Foundation project (Grant No.: PG/22/11121). M.B.: MRC Clinician Scientist Fellowship (Grant No.: 1519588). A.S.: Kidney Research UK (project grant) and Medical Research Council [1. Clinician Scientist Fellowship Grant No.: G0802829] [2. Centenary Early Career Award]. M.G.: A University of Bristol MRC IAA. R.R.: Kidney Research UK John Feehally/Stoneygate project grant (Grant No.: JF‐S/RP/2015/10).

## ETHICAL APPROVAL

All animal work was carried out under Home Office Project Licence PP2775164, with local University Animal Welfare and Ethics Review Body approval.

## Supporting information



Supporting Information

## Data Availability

All summary data are included within the manuscript. There are no large datasets, and all raw data can be provided on request.
