# Supplementary material for: Heparanase inhibition prevents glycocalyx damage and albuminuria in experimental minimal change disease
Source: Clin Transl Med. 2026 May 31;16(6):e70706. doi: 10.1002/ctm2.70706 (PMC13240007; doi:10.1002/ctm2.70706)
Supplement: Supplementary file 1 — Supporting Information [file CTM2-16-e70706-s001.docx]

**Supplementary figure 1. MAb 5-1-6 targets podocytes specifically to induce proteinuria.** (A) Schematic to show experimental design of female Lewis rats given mAb 5-1-6 to induce anti-nephrin antibody nephropathy (AN-Ab) or left untreated. Kidneys were removed from sham (i) or AN-Ab (ii) rats on day 6 post-treatment and flash frozen for immunofluorescence or sieved for QPCR analysis. Co-localisation immunofluorescence was performed with AF568 secondary antibody to bind to mAb 5-1-6 (red), Dapi (nuclear stain, blue) and anti-podocin (green, Bi-ii) or WT-1 (green, Ci-ii). WT1 number per glomerulus was quantified (Ciii, n=7 and 7). cDNA was extracted from sieved glomeruli and nephrin expression was quantified relative to GAPDH (2-^^^CT^), unpaired t test, n=4 (D). Urine collected from these rats at days 0, 3 and 5 were analysed for urine albumin:creatinine ratio (ACR), one way ANOVA, Bonferroni post hoc tests indicated, n=4 (E). *=p<0.05. *=p<0.05, **=p<0.01, ***=p<0.001.

**Supplementary figure 2. Heparanase inhibition did not impact nephrin distribution**. Female Lewis rats were induced with anti-nephrin associated nephropathy (AN-Ab) or left untreated. Of those AN-Ab rats, half were given heparanase inhibitor (HI), OVZ/16-35, from day 1 (daily) and half were given vehicle. (A) Representative images of immunofluorescence for anti-nephrin is shown, for each group. (B) A measure of nephrin linearity was quantified. Distribution per rat is shown (Bi, Kruskal-Wallis test, p<0.01) with Dunn’s multiple comparisons test, n=4, p<0.05) and of all glomeruli (Bii).


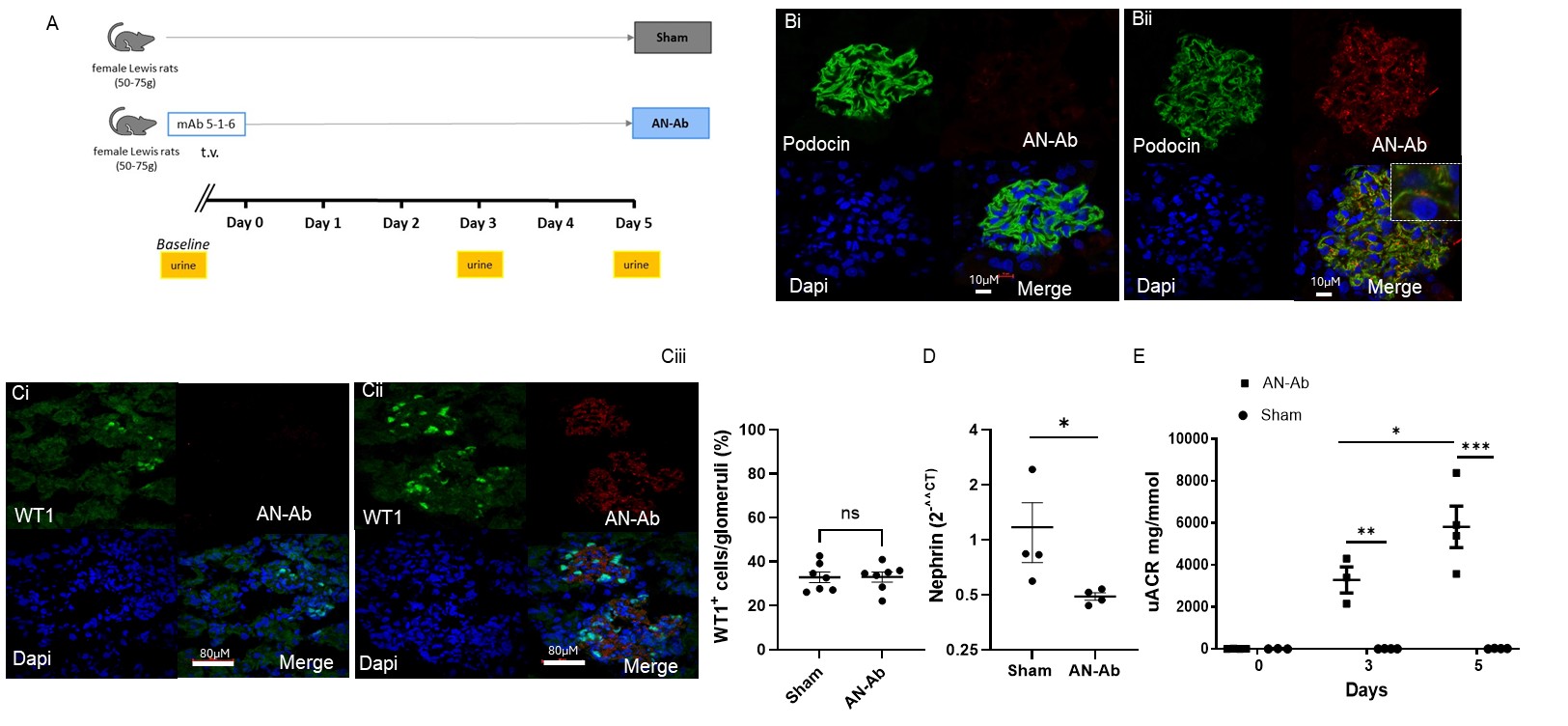
Supplementary figure 1.


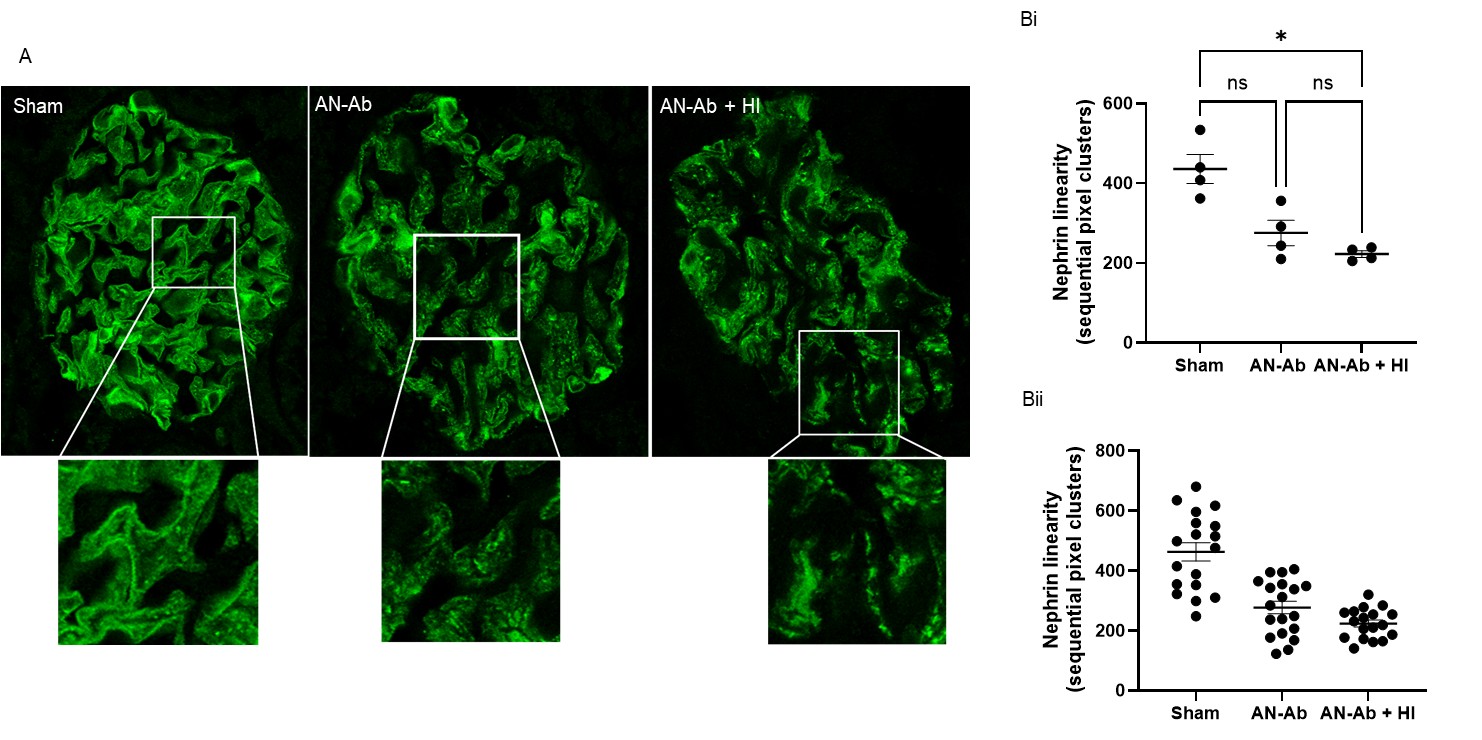


Supplementary figure 2.
